# Fabrication of multifunctional ZnO@tannic acid nanoparticles embedded in chitosan and polyvinyl alcohol blend packaging film

**DOI:** 10.1038/s41598-024-68571-9

**Published:** 2024-08-09

**Authors:** Maha Sultan, Ahmed Youssef, Rasha A. Baseer

**Affiliations:** 1 Packaging Materials Department, Chemical Industries Research Institute, National Research Centre, 33 El Bohouth St. (Former El Tahrir St.), Dokki, P.O. 12622, Giza, Egypt; 2 Department of Polymers and Pigments Technology, Chemical Industries Research Institute, National Research Centre, 33 El Bohouth St. (Former El Tahrir St.), Dokki, P.O. 12622, Giza, Egypt

**Keywords:** Zinc oxide, Tannic acid, UV-shielding, Antioxidant, Chitosan, Polyvinyl alcohol, Materials science, Chemistry, Materials chemistry, Polymer chemistry

## Abstract

The current study explores biodegradable packaging materials that have high food quality assurance, as food deterioration is mostly caused by UV degradation and oxidation, which can result in bad flavor and nutrition shortages. Thus, new multifunctional zinc oxide nanoparticles/tannic acid (ZnO@TA) with antioxidant and antibacterial activities were incorporated into polyvinyl alcohol/chitosan (PVA/CH) composite films with different ratios (1%, 3%, and 5% based on the total dry weight of the film) via a solution blending method in a neutral aqueous solution. Additionally, ZnO nanoparticles have unique antibacterial mechanisms through the generation of excessive reactive oxygen species (ROS) that may lead to intensify pathogen resistance to conventional antibacterial agents. Thus, minimizing the negative effects caused by excessive levels of ROS may be possible by developing unique, multifunctional ZnO nanoparticles with antioxidant potential via coordination bond between tannic acid and ZnO nanoparticles (ZnO@TA). ZnO@TA nanoparticles were examined using Fourier-transform infrared** (**FTIR), X-ray diffraction (XRD), and scanning electron microscopy (SEM). The effect of the incorporation of ZnO@TA nanoparticles on the barrier, mechanical, thermal, antioxidant, antimicrobial, and UV blocking characteristics of chitosan/polyvinyl alcohol (ZnO@TA@CH/PVA) films was investigated. The lowest water vapor and oxygen permeability and the maximum antioxidant capacity% are 31.98 ± 1.68 g mm/m^2^ kPa day, 0.144 ± 5.03 × 10^–2^ c.c/m^2^.day, and 69.35 ± 1.6%, respectively, which are related to ZnO@TA(50)@CH/PVA. Furthermore, ZnO@TA(50)@CH/PVA film exhibits the maximum UV shielding capacity of UVB (99.994). ZnO@TA(50) @PVA/CH films displayed better tensile strength and Young`s modulus of 48.72 ± 0.23 MPa and 2163.46 ± 61.4 MPa, respectively, than the other film formulations. However, elongation % at break exhibited the most reduced value of 19.62 ± 2.3%. ZnO@TA@CH/PVA film exhibits the largest inhibition zones of 11 ± 1.0, 12.3 ± 0.57, and 13.6 ± 0.57 mm against *Staphylococcus aureus*, *Aspergillus flavus*, and *Candida albicans*, respectively. In accordance with these results, ZnO@TA@CH/PVA films could be utilized for food preservation for the long-term.

## Introduction

Perishable products have a short shelf life due to spoilage and oxidation caused by microbial activity and metabolic/enzymatic processes. This can lead to a decrease in product quality and safety, as well as high economic losses, increased costs, and a higher risk of consumer poisoning on a commercial scale^1^. Currently, active packaging based on natural polymers seeks to reduce environmental risks brought on by non-biodegradable food packaging waste while also providing food that is safe and of high quality with a longer shelf life^2^. This creative packaging not only serves as a barrier but also as a preservation technique^3^.

Chitosan has gained extensive interest because of its non-toxicity, antibacterial, and antifungal properties. Chitosan is therefore regarded as an ideal material for the creation of food-grade films. On the other hand, pure chitosan is only soluble in acidic conditions, has weak mechanical characteristics, and loses its antibacterial action at pH values higher than 6.5. The most effective way to overcome these problems is by combining chitosan with other polymers, such as gelatin, polylactic acid (PLA), and polyvinyl alcohol (PVA)^4^.

PVA is a synthetic polymer that has good miscibility and film-forming properties^5^. It is non-toxic and biodegradable. PVA films have excellent oxygen barrier characteristics, high strength, and good resilience to acid and alkali. PVA's compatibility allows it to be utilized to be blended with chitosan through the creation of a hydrogen bond, enhancing the material's mechanical properties^6,7^. Recent developments in chitosan/polyvinyl alcohol (PVA) blend food packaging films are the main topic of recent studies. It addresses applications in food preservation as well as molecular structure, characteristics, and performance-enhancing techniques. The blend is a viable option for biodegradable films due to its good compatibility and intermolecular interactions^8,9^.

Multifunctional polyvinyl alcohol/chitosan composite films with pH-sensitive, antibacterial, antioxidant, and UV-barrier capabilities have been developed by Ahmed A. Oun et al. in 2022. The cellulose nanocrystals (CNCs), grapefruit seed extract (GSE), and aronia, or black chokeberry (Aronia melanocarpa) were utilized. When CNCs were added, the PVA/CH composite film's tensile strength improved by 74%, while the addition of GSE boosted the film's flexibility by 75%. The composite film constructed from PVA and CH-A (aronia extract) exhibited strong antioxidant activity and a notable color shift at varying pH levels. In addition, the PVA/CS-G (GSE) exhibited the greatest antibacterial action against *Listeria monocytogenes* and *Escherichia coli.* With the best UV-barrier (95.5%), maximum antioxidant activity (95%), pH sensitivity, lowest water vapor permeability (WVP), and desired mechanical properties, the PVA/CS-CGA composite film was reinforced with CNCs/GSE/Aronia extract^10^.

The epidemic of infectious diseases brought on by various dangerous bacteria has scientists looking for novel antibacterial treatments. Nowadays, because of their distinct chemical and physical characteristics and high surface area to volume ratio, nanoscale materials have become new and innovative antibacterial agents^11,12^.

ZnO NPs have been the subject of current research among other nanomaterials because of their characteristics and potential uses in the future. ZnO NPs were added to biopolymer packaging materials, which significantly increased the antibacterial action against foodborne pathogens and extended shelf life of food. ZnO is used as a multipurpose ingredient in food packaging films due to its antibacterial action, UV-blocking characteristics, and reinforcement of nanocomposites aptitudes^13,14^.

Zinc oxide nanoparticles (ZnO NPs) produced from food industry by-products have been incorporated into chitosan in different concentrations. The composites' properties changed from brittle to flexible, with decreased tensile strength and Young modulus and increased elongation at break. This preserved the good barrier features while also favoring the permeability of water vapor and oxygen. The addition of ZnO nanoparticles had no significant impact on the optical characteristics.^15^.

ZnO nanoparticles have unique antibacterial mechanisms that allow them to interact with the bacterial surface or core immediately upon contact. On the other hand, excessive reactive oxygen species (ROS) produced by ZnO nanoparticles may lead to genomic instability, membrane protein damage, and cell membrane disintegration, all of which can start or intensify food borne pathogen resistance to conventional antibacterial agents. Thus, minimizing the negative effects caused by excessive levels of ROS may be possible by developing unique and multifunctional ZnO nanoparticles with antioxidant activity^16,17^.

Tannic acid (TA) is gallic ester of D-glucose in which the carbohydrate's hydroxyl groups are completely esterified with gallic acid dimers. Because of its numerous phenolic groups that can interact with biological macromolecules, TA is known for its antioxidant ability^18^. Su Jin Lee et al.^19^ have prepared multifunctional chitosan/tannic acid composite films with better antioxidant, anti-UV, and antimicrobial properties for active food packaging. High transmission rates were indicated by the pure CH film's WVTR and OTR, which were 956 g/m^2^⋅day and 0.39 cc/m^2^⋅day, respectively. Conversely, the transmission rates of the CH-TA composite films were noticeably lower than those of the pure CH film.

Compared to the pristine CH film, the CH-TA composite films demonstrated remarkably stronger antioxidant activity. The pure CH and CH-TA films exhibited antioxidant (DPPH radical scavenging) activity of 12.7 ± 1.7% and 90.0 ± 0.8%, respectively. The antibacterial activity of the pure CH film against *S. aureus* and *E. coli* was extremely poor. On the other hand, because of TA's superior antibacterial qualities, the CH-TA composite films demonstrated significant antibacterial activity against these two microorganisms^19^. Quercetin was added to three distinct polymers, namely carboxymethyl cellulose, gelatin, and poly(lactic acid), to create three functional packaging films by Parya Ezati and Jong-Whan Rhim (2021). The addition of quercetin totally stopped the biopolymer films' ability to transmit UV light. The films containing quercetin exhibited antibacterial and antioxidant properties, dependent on the quercetin release rate. Significant inhibitory function was demonstrated by the CMC/quercetin and gelatin/quercetin films against *Escherichia coli* and *Listeria monocytogenes*^1^.

Thus, this study examined for the first time a straightforward surface modification method that uses tannic acid to functionalize ZnO nanoparticles with antimicrobial activity. A novel multifunctional nanoparticle with antibacterial, antioxidant, and UV properties is described in this work. Through coordination bond of tannic acid (TA) with ZnO nanoparticles, ZnO@TA nanoparticles were created. ZnO@TA nanoparticles were examined using FTIR, TEM, XRD, particle size, and Zeta potential. The prospective impact of incorporating different concentrations of ZnO@TA into CH/PVA films on water vapor and oxygen permeability, water contact angles, film solubility, mechanical, thermal, antioxidant, and antimicrobial properties. The produced films were investigated via FTIR, XRD, SEM (EDAS/mapping) and UV blocking capacity.

## Materials and methods

Zinc acetate dihydrate 98% was purchased from Merck. Chitosan medium molecular weight with deacetylation percentage of 75–85% deacetylated, poly (vinyl alcohol) (PVA) molecular weight 89.000–98.000 (+ 99%-degree hydrolysis) were acquired from Merck. Tannic acid (Mw 1701.2 g/mol) was purchased from Sigma Aldrich. The solvent of chitosan was 98% acetic acid which purchased from a local company of El Naser (Egypt).

### Preparation of ZnO nanoparticles

ZnO nanoparticles were prepared as described. A stock solution of (0.1 M) zinc acetate dihydrate was vigorously stirred into methanol (50 mL). The pH of the mixture was then maintained at 8 by adding (0.2 M) NaOH in methanol (25 mL). The mixture was sealed in a Teflon-lined stainless-steel autoclave at 80 °C for 10 h. The white precipitate was washed with methanol, filtered, and then dried in a vacuum oven at 60 ◦C for 6 h.^16^.

#### Preparation of ZnO@tannic acid nanoparticles

A wet chemical process with tannic acid was used to provide ZnO nanoparticles with antioxidant functionality, as follows. Subsequently, 20 mg of ZnO nanoparticles were dispersed in 1 mL of EtOH. Then the above solution was poured to a solution of tannic acid/EtOH (2.08 × 10^−5^ M), vigorously stirred for 24 h. The resulted pale brown precipitate was washed several times in EtOH and dried at 60 ◦C^17^.

#### The average particle size and Zeta-potential of ZnO@TA

The average particle size and Zeta-potential of ZnO@TA was determined with the aid of a dynamic light scattering (DLS) PS analyzer (ZetaSizer Nano ZS ZEN3600 from Malvern Instrument; Particle Sizing Systems, Inc. Santa Barbara, CA), every measurement was done at 25°C. The samples were serially diluted 100 times using deionized water.

### Fabrication of ZnO/TA@ CH/ PVA films

CH/PVA film was used as a control, and (0.5 g) of PVA was dissolved in 50 mL of hot, deionized water while being stirred. Chitosan solution (0.5 g/50 mL of 2% acetic acid) was added to PVA solution under stirring with addition of 0.1 g glycerol. After ultrasonic treatment, the mixed solution was then poured into Teflon dishes and let to air dry. ZnO/TA were added with different loadings (1%, 3%, and 5%) based on CH/PVA (1 g) which are equivalent to 10 mg, 30 mg, and 50 mg ZnO/TA, and solid content of all films was kept constant to get a constant thickness (0.166 ± 5.77 × 10^−3^ mm) as illustrated in Table 1. The prepared films are referred to CH/PVA, ZnO@TA(10)@CH/PVA, ZnO@TA(30)@CH/PVA, and ZnO@TA(50)@CH/PVA.

**Table 1:** Compositions of CH/PVA and ZnO@TA@CH/PVA films.

Sample	Chitosan (g)	Polyvinyl alcohol (g)	ZnO@TA (g)	Glycerol (g)
CH/PVA	0.5	0.5	0	0.1
ZnO@TA(10)@CH/PVA	0.495	0.495	0.01	0.1
ZnO@TA(30)@CH/PVA	0.485	0.485	0.03	0.1
ZnO@TA(50)@CH/PVA	0.475	0.475	0.05	0.1

### Characterization

#### Fourier-transform infrared (FTIR)

The chemical structures of ZnO NPs, TA, ZnO@TA, CH/PVA, and ZnO@TA@CH/PVA films were determined by Fourier Transformation Spectroscopy (FTIR) spectra at room temperature using the Attenuated Total Reflection (ATR) unit attached with FTIR-Vertex 70 Bruker, Germany, in the range of 4000–400 cm^‒1^.

#### X-ray diffraction (XRD)

X-ray diffractions of ZnO NPs, TA, ZnO@TA, CH/PVA, and ZnO@TA@CH/PVA were investigated using a Bruker diffractometer (Bruker D 8 advance target). CuKα radiation source with secondly monochromator (λ = 1.5405 Ǻ) at 40 kV and 40 mA was used. 0.2 min^−1^ was the scanning rate for phase identification and line broadening profile analysis.

Using the Scherer equation (Eq. 1) and the diffraction intensity of the (101) peak, it is simple to determine the average grain size of ZnO NPs as a function of peak width (described as the full-width at half maximum peak intensity (FWHM)), peak location, and wavelength.

1 $$D= \frac{0.89 \lambda }{\beta \text{Cos}\theta }$$

where λ is the wavelength (Cu Kα), β is the full width at the half- maximum (FWHM) of the ZnO (101) line and θ is the diffraction angle^20^.

#### Transmission electron microscopy (TEM) analysis

Transmission electron microscopy (TEM) analysis was conducted using JEM-10OCXII TEM (Japan) at 120 kV. The freshly-prepared sample solutions were dropped on carbon coated copper grid to obtain a highly thin film. The ZnO NPs were ready to be investigated after 15 min.

#### Morphology studies (SEM)

SEM Model Quanta 250 FEG (Field Emission Gun) attached to EDAX Unit (Energy Dispersive X-ray Analyses), with accelerating voltage 30 KV, magnification 14× up to 1,000,000, and resolution for Gun.1n).

#### Water vapor and oxygen permeability (WVP&OP)

The water vapor transmission rate (WVTR) was estimated using a GBI W303 (B) Water Vapor Permeability Analyzer (China) using the cup method. According to a standard (ASTM E96) as the total amount of water vapor transported over a unit area in a unit of time under controlled temperature (38 °C) and humidity (4%) conditions. In addition, the gas transmission rate (OTR) was evaluated using a N530 Gas Permeability Analyzer (China) in accordance with ASTM D1434-82 (2003).

The slope (WVTR in g day^−1^) was estimated from equation (Eq. 2).

2 $${\text{WVTR}}={\text{L}}\times \, \frac{1}{A}$$

*L* is the slope and the film area is indicated by *A* (m^2^).

WVP (g mm/m^2^ kPa^−1^ day^−1^) was calculated using Eq. (3)

3 $$WVP=L\times \frac{\text{WVTR}}{\Delta Pressure}$$

*L* is the film`s thickness (mm) and $\Delta Pressure$ is the partial pressures of water vapor in saturated air with 100% relative humidity and 38 °C and air ($\Delta Pressure$ = 5.942 kPa).

The OPTR (Eq. 4) and OP (Eq. 5) were calculated.

4 $${\text{OPTR}}=\frac{Slope}{A}$$

5 $${\text{OP}}=OPTR \times \frac{f}{\Delta P} \,$$

where *A* is the film area (m^2^), $\Delta P$ is the gas partial pressure difference (0.02308 atm, at 25 °C), and *f* is thickness of the film (m)^21,22^.

#### Solubility percentage

Solubility was calculated using Eq. (6). Film specimens were sliced (2 cm × 2 cm) and weighted (accuracy 0.0001 g), then dried in an oven at 7 °C for 24 h to determine the initial dry mass (M1). The samples were placed in a Petri dish containing 30 mL of water for 24 h at room temperature (25 ± 2 °C) allowing water swelling. Finally, the remaining film specimens were dried in the oven under the same circumstances as previously to determine the final dry mass (M2). For each film sample, two measurements were conducted, and results were expressed as a percentage of the average of the two determinations.^23^

6 $$Solubility \left(\text{\%}\right)= \frac{ {\text{M}}_{1} - {\text{M}}_{2}}{{\text{M}}_{1}} \times 100$$

#### Water contact angle measurements (WCA)

The water contact angle of the generated CH/PVA and Zn@TA@CH/PVA films has been determined utilizing a Theata Optical Tensiometer (Data Physics, Model OAC 13EC) (Germany). To calculate the contact angle values, data physics software used the Young fitting procedure on water drop photos collected at the contact point.

#### Mechanical properties

The thickness of produced films was measured using a thickness dial gauge at three distinct spots, and the mean values were determined. Using electronic universal material testing equipment (Instron 34SC-5 Universal Machine, UK) with a load cell of 5KN, the mechanical characteristics of the films' tensile stress and strain were evaluated. Before testing at a cross head speed of 10 mm per minute, the samples were divided into 80 mm × 20 mm pieces and kept at 23 ± 2 °C and 50 ± 5% relative humidity (RH) for 48 h. The tensile speed was 20 mm/s. Each sample was assessed at least three times, and the average result was noted. Using formula (7), the tensile strength at break (TS) was determined.

7 $$TS (\text{MPa})= \frac{ {\text{F}}_{\text{max}} }{{\text{d}}\times {\text{w}} }$$

where *F*_max_ is the maximum tension (N),* d* and *w* are the thickness (mm) and width (mm) of the film, respectively^24^.

Elongation % at break (*EAB* %) was calculated as formula (8).

8 $$EAB \left(\%\right)= \frac{ {\text{L}}_{1} - {\text{L}}_{0}}{{\text{L}}_{0} } \times 100$$

#### DPPH radical scavenging ability

The antioxidant capacity of CH/PVA and ZnO@TA@CH/PVA films of different ZnO@TA loadings was assessed using the 2-diphenyl-1-picrylhydrazyl hydrate (DPPH) radical scavenging assay (DPPH). A total of 24 mg of DPPH were dissolved in 100 mL of methanol for making the stock solution. Filtration of DPPH stock solution using methanol yielded a usable mixture with an absorbance of around 0.973 at 517 nm^25^. In brief, 4 ml of a 100 mM DPPH methanol solution was added to 20 mg of the film. For one hour, the reaction was kept in the dark. At 517 nm, the absorbency of the reaction solution was measured^26^.

For the ABTS assessment, an ABTS solution was prepared by adding 7 mM of ABTS to 2.4 mM of potassium persulfate solution in a 1:0.5 (v/v) ratio and preserved in the dark for 12–16 h. 7 mM of ABTS to 2.4 mM of potassium persulfate solution in a 1:0.5 (v/v) ratio and preserved in the dark for 12–16 h. The ABTS solution was diluted to attain an absorbance of 0.7 (± 0.1) at 734 nm. Similarly, to the DPPH test, about 20 mg of the film sample was immersed in 4 mL of the ABTS solution in the dark for 60 min. The film sample was subsequently picked out to measure the absorbance of the ABTS solution. A pure ABTS solution was used as the control, and the radical scanning activity was analyzed based on the difference in the absorbance of the solution at 734 nm (n = 5). The antioxidant activities of the films were calculated as follows:

Accordingly, DPPH radical scavenging activity was obtained (Eq. 9):

9 $$DPPH \,scavening \,ability \,text{ \,\% }=\frac{ {\text{A}}_{0}{-\text{ A}}_{\text{S}} }{ {\text{A}}_{0} }\times 100$$

where, *A*_o_ is the absorbance of the pure DPPH or ABTS solution and *A*_s_ is the absorbance of the same solution after the immersion of the film^19,27^.

#### Ultraviolet blocking analysis

The UV-blocking features of CH/PVA and ZnO@TA@CH/PVA films were evaluated by assessing their absorbance and total transmittance from 200 to 2500 nm (UV–Vis-NIR spectrum, Japan). The T (UVA) and T (UVB) of CH/PVA and ZnO@TA@CH/PVA films were estimated using the equations listed below (Eqs. 10 and  11), respectively^28,29^.

10 $$T\left(UVA\right) \, {\%}=100- \frac{{\int }_{320}^{400}{T}_{\lambda } \times d\lambda }{{\int }_{320}^{400}d\lambda }$$

11 $$T\left(UVB\right)\, {\%}=100- \frac{{\int }_{280}^{320}{T}_{\lambda } \times d\lambda }{{\int }_{280}^{320}d\lambda }$$

Where, T_λ_ is the transmittance of TCC/DAC-TH films of the light at the wavelength.

#### Thermal analysis

Thermo gravimetric assessment have been utilized to evaluate the thermal stability of CH/PVA and ZnO@TA@PVA/CH films (Universal V4.5A TA Instruments SDT Q600 V20.9 Build 20) under nitrogen. Based on information about weight loss, kinetic investigations were done using the TG curve. The thermodynamic parameters of deterioration were identified. A chemical reaction's rate is commonly assumed (Eq. 12).

12 $$Rate=dc/dt={kc}^{n}$$

where, $c$ is the weight of the residual constituents at time *t* for each fragmentation step, and $n$ is the order of the reaction. Equation (13) was utilized to provide further explanation:

13 $$k=-\frac{dw}{dt}={k\left({\text{w}}_{\text{t}}{ - \, {\text{w}}}_{\infty}\right)}^{n}$$

where, *w*_*t*_ is the sample mass at time *t* and *w*_∞_ is the mass at the end of the fragmentation process. Applying Arrhenius equation to obtain the Eq. (14).

14 $$logk={\text{log}(\frac{dw}{dt})/\left({\text{w}}_{\text{t}}{ -\text{ w}}_{\infty}\right)}^{n}= logA-\frac{\Delta { \text{E }}_{\text{a}}}{RT}$$

*A* is the Boltzmann constant, *R* is the general gas constant (8.314 Joules/deg. mole), *T* is the absolute temperature (Kelvin), and the *E*_a_ is activation energy. The reaction rate $(-dw/dt)$ could be modified into Eq. (15):

15 $$-\frac{dw}{dt}= \frac{ {\text{w}}_{1}{ -\text{ w}}_{2} }{ {\text{t}}_{2}{ -\text{ t}}_{1} }$$

where, *w*_1_ and *w*_2_ are residual amount at times* t*_1_ and *t*_2_, respectively. The least square approach and *n* values from 0.0 to 3.0 with 0.5 increments were were used to determine the highest R2, lowest standard error for each *n* and total activation energies could be estimated^30,31^*.*

#### Antimicrobial properties

The agar disc diffusion procedure or the Kirby-Bauer disc diffusion process was exploited to assess the inhibitory investigation of PVA/CH and ZnO@TA@PVA/CH films with different ZnO@TA contents against *Staphylococcus aureus, Aspergillus flavus,* and *Candida albicans.*

#### Statistical analysis

Each experiment was repeated at least three times. Using the "SPSS" application, the results were presented as mean standard deviation (SD). The results were subjected to statistical analysis using the Duncan test to identify variations among treatments at a significance level of 0.05.

## Results and discussion

**Figure 1:**
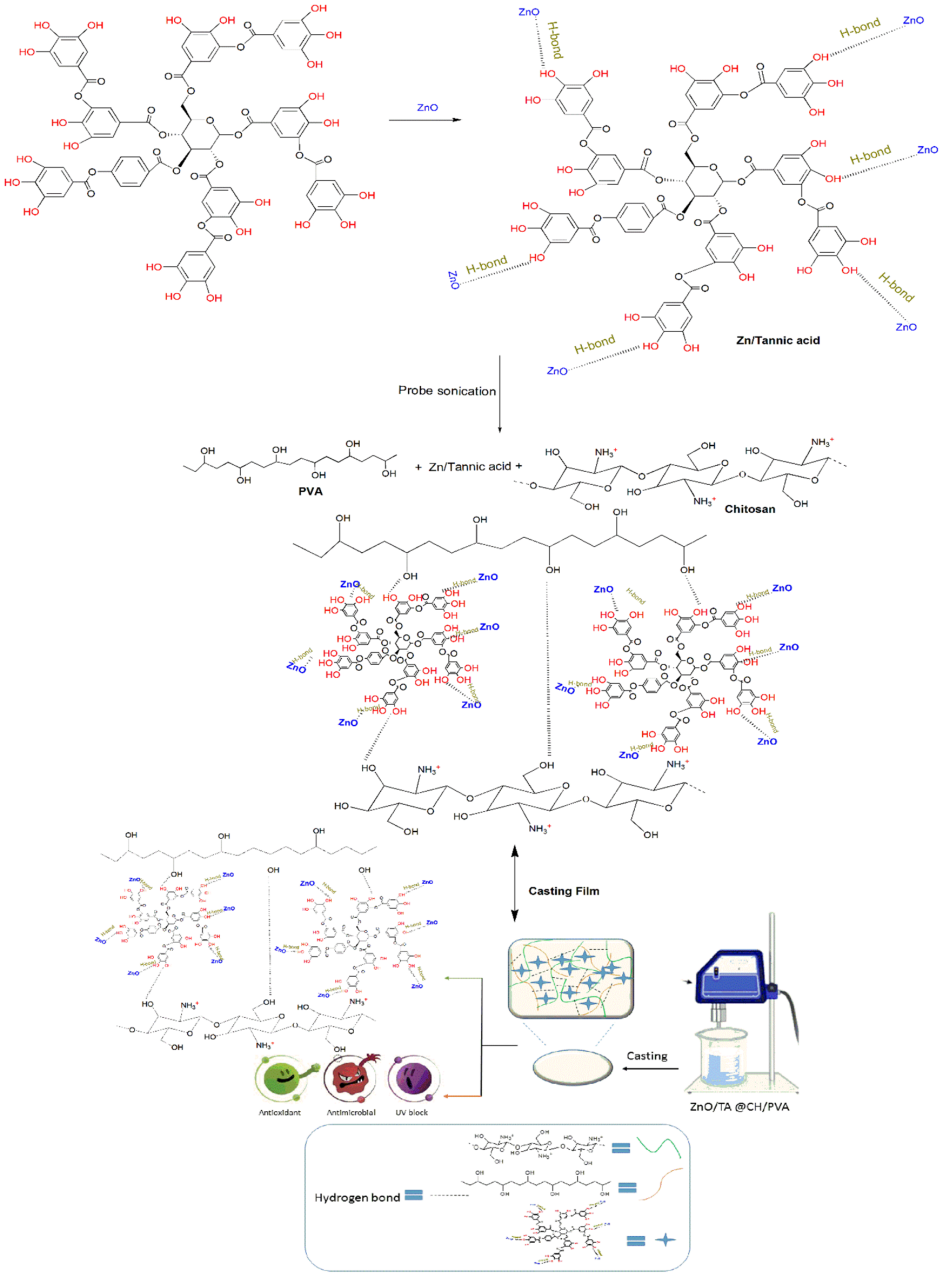
A schematic diagram of suggested mechanism of ZnO@TA formation and construction of ZnO@TA@CH/PVA films.

The multifunctional ZnO@TA@PVA/CH films with antibacterial, antioxidant, and UV blocking properties are as-prepared as illustrated in Fig. 1. The possible mechanism of surface modification on ZnO NPs with TA acid involves two proposed mechanisms through formation of hydrogen bonding between –OH of tannic acid with ZnO NPs surface and formation of a π-complex between the surface of ZnO NPs and tannic acid containing phenyl groups through sharing of π-electrons of phenolic rings present in the tannic acid with 4S orbital on Zn^+2^ via a coordination bonding^32^.

### FTIR

**Figure 2:**
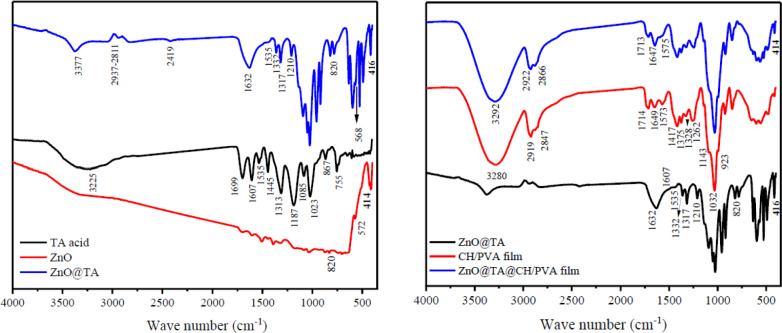
ZnO NPs, TA, ZnO/TA, CH/PVA, and ZnO/TA@PVA/CH.

Figure 2 illustrates the FTIR spectrum to determine the specific functional groups or chemical bonds for ZnO NPs, TA, ZnO@TA, CH/PVA, and ZnO@TA@CH/PVA film.

For ZnO NPs, the absorption peak at 414, 572, and 820 cm^-1^ corresponds to metal–oxygen (ZnO stretching vibrations) vibration mode^33–36^.

For FTIR spectra of tannic acid show a significant absorption of about 3480–3000 cm^−1^ with a wide and strong band centered at 3225 cm^−1^. This band is corresponded to the broad and strong H-bonded hydroxyl groups (O–H) as well as the C–H bond (aromatic medium). Tannic acid consists of aromatic esters due to the signal properties of carbonyl groups C=O stretching (1699 cm^−1^) and C–O (1313–1187 cm^−1^). The in-plane bending of C–O–H group occurs at 1607–1535 cm^−1^, and the out-of-plane bending was assigned to 872 cm^−1^^37^. Bands at 1607–1445 cm^−1^ associated with C–C of aromatic compounds. Various peaks in the 1085–755 cm^−1^ associated with substituted benzene ring^38^.

For FTIR spectrum of ZnO@TA, the characteristic bands of the ZnO@TA nanoparticles and pristine TA were remarkably comparable, resembling the conventional peaks of TA. It could be claimed that the interaction is pronounced as a π-complex between tannic acid containing phenyl groups and the surface of ZnO NPs. Moreover, the characteristic peaks of aromatic C=C of tannic acid at 1445 cm^−1^ and 1607 cm^−1^ were disappeared, indicating the participation of the C=C group in the creation of the coordination complex. This could be due to the interaction of π-electrons of phenolic rings in tannic acid with the 4S orbital on Zn^+2^ via a coordination link, generating a sandwich-like structure^32^. A significant shift in the band assigned to the hydroxyl groups (O–H) H-bonded and C–H (aromatic medium) at 3225 cm^−1^ to 3377 cm^−1^ with somewhat sharpness due to the proposed coordination and hydrogen bonding between ZnO NPs and TA. The out-of-plane bending was assigned to 867 cm^−1^ shift to 820 cm^−1^. These findings indicate that the hydroxyl group of TA could form hydrogen bonding with ZnO^39^. The absorption peak at 414 cm^−1^ which corresponds to metal–oxygen (ZnO stretching vibrations) vibration mode is strongly observed in ZnO@TA.

For the virgin CH/PVA IR curve has a substantial peak at 3291 due to the overlapping of the -OH and NH_2_ groups of PVA and CH, besides a peak at 2861 related to –CH group^28^. These peaks are signatures of polysaccharides and can be witnessed in other polysaccharide spectra^40^. Other peaks at 1649 are due to the C=N amine bond of CH, and at 1561 to NH_3_^+^, which is formed by NH_2_ deformation in acidic conditions. CH_2_ bending and CH_3_ symmetrical deformations have been verified with bands at 1423 and 1375 cm^−1^, respectively. The absorption peak at 1143 cm^−1^ indicates asymmetric stretching of the C–O–C bridge. The peaks at 1032 and 923 cm^−1^ indicate C-O stretching.^41^ which is characteristic of saccharide structure of chitosan.

FTIR spectrum of ZnO@TA@CH/PVA film showed the same spectrum of CH/PVA film with minor shift. The addition of ZnO@TA into CH/PVA causes a minor shift from 3280 to 3292 cm^−1^ due to hydrogen bonding between CH/PVA and ZnO@TA. The absorption peaks which correspond to metal–oxygen (ZnO stretching vibrations at 414 cm^−1^) of ZnO@TA is powerfully detected in FTIR spectrum of ZnO@TA@CH/PVA indicating the inclusion of ZnO@TA.

### X-ray diffraction analysis

**Figure 3:**
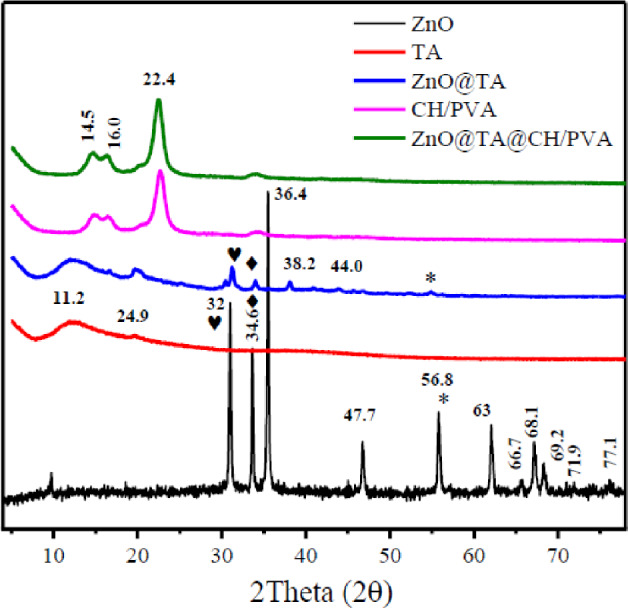
XRD patterns of ZnO NPs, tannic acid (TA), ZnO@TA, CH/PVA, and ZnO@TA@CH/PVA films.

Figure 3 verified XRD patterns of ZnO NPs, TA, ZnO@TA, CH/PVA, and ZnO@TA@CH/PVA. The ZnO NPs exhibited 2Theta at 32.0°, 34.6°, 36.4°, 47.7°, 56.8°, 62.0°, 66.7°, 68.1°, 69.2°, 71.9°, and 77.1°, respectively that were corresponding to diffraction planes (100), (002), (101), (102), (110), (103), (200), (112), (201), (004), and (202), respectively. XRD spectrum profile of ZnO verified hexagonal wurtzite phase^42^.

The average grain crystallite size Scherer equation of ZnO NPs were estimated utilizing Scherer equation and have an estimated value equaled 1.727 nm. No noticeable peaks of crystalline phase were observed in the XRD patterns of TA, but characteristic two signals (2θ = 11.2° and 24.9°), representing the amorphous nature of TA.

The XRD pattern of the ZnO@TA showed crystalline diffraction peaks with minor shift at 2θ = 32.0°, 34.6°, 38.2°, 44.0°, and 56.8° were observed clearly which are indexed by diffraction planes (100), (002), (101), (102), and (110) of ZnO NPs (Li, et al. 2016).

The X-ray diffraction profiles of CH/PVA and ZnO@TA@CH/PVA films are shown in Fig. 3. The XRD profile of CH/PVA and ZnO@TA@CH/PVA films are very similar. The CH/PVA film diffractogram exhibits peaks in 2 Theta = 16.9°, 23.2°, and 34.6° where amorphous diffraction peaks at 2 Theta = 23.2° is indicative of the blending of CH/PVA^43^. The 2θ value at 19.43° revealed the crystal structure of PVA, which was found across all diffraction patterns of the generated composite films was shifted to 22.4° may be attributable to blending with chitosan. The specific signals of ZnO@TA are not observed in diffractogram of ZnO@TA@CH/PVA indicating that trace amount of ZnO@TA was altered when added to CH/PVA matrix. In order to determine the crystallinity alteration of CH/PVA film when ZnO@TA was added, the crystallinity index of ZnO@TA@CH/PVA was calculated by the following equation (Eq. 16):

16 $${\text{X}}_{\text{i}} \%=\frac{{\text{A}}_{\text{C}}}{{\text{A}}_{\text{C}}+{\text{A}}_{\text{a}}} \, \times \text{100 }$$

where, *A*_c_ and *A*_a_ are crystalline area and amorphous, respectively^44^.

The crystallinity index % of CH/PVA was 67.07% while ZnO@TA@PVA/CH showed a slight increase with inclusion of ZnO@TA given 71.44%.

### Transmission electron microscopy analysis

**Figure 4:**
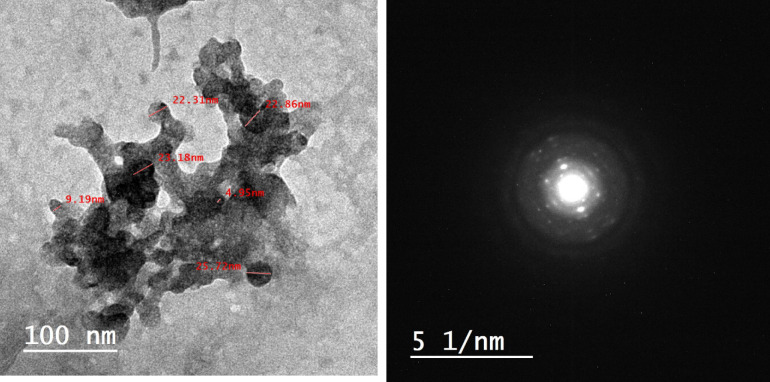
TEM and selected electron diffraction area of ZnO.

TEM reveals the microscopic structural characteristics of ZnO NPs. Figure 4 shows TEM images of ZnO NPs, where ZnO NPs appear to have a uniform and spherical shape with particle size within the range of 4.95–25.72 nm. These results were consistent with that reported by Razieh Galal et al. (2010)^45^. Figure 4 depicts the selected electron diffraction area of ZnO NPs revealing the nano-crystalline structure of ZnO NPs which accords with XRD analysis.

### Particle size and zeta potential measurements

**Figure 5:**
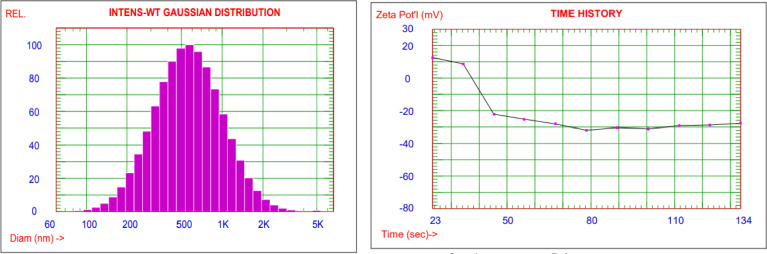
Particle size distribution of ZnO@TA NPs.

Figure 5 shows the Zeta Potential and particle size distribution of ZnO@TA. ZnO@TA NPs have a mean hydrodynamic diameter of 663.8 ± 0.57 nm. Tannic acid aggregation on ZnO NPs arose due to the impact of acidic medium caused by TA molecule^46^, results in the formation of ZnO@TA complex could be the cause of the increased mean hydrodynamic diameter (MHD) of ZnO@TA. Abebe Belay et al. (2015) recently noticed a similar effect. When caffeic acid was added to the ZnO NPs, it was observed that the MHD of the ZnO@caffeic acid NPs increased. When the concentration of ZnO NPs was 7.55 × 10^–6^ M, the MHD of ZnO/caffeic acid was 295 nm. When ZnO NPs concentration was 7.55 × 10^–6^ and 1.30 × 10^–5^ M, respectively, the MHD of ZnO@caffeic acid increased to 396 and 955 nm^47^.

The Zeta Potential, which represents the NPs' surface charge, is one significant factor which influences particle stability. If the electric charge on the particle surface is larger, the NPs are not likely to assemble. The Zeta Potential numerical value of ZnO@TA is − 27.83 mV. This indicates ZnO@TA NPs complex are well stabilized due to repulsive forces that prevent aggregations upon aging. The recognized range of the Zeta Potential to provide adequate stability in solution is − 30 to − 20 mV or + 20 to + 30 mV, according to Mojtaba Taghizadeh et al.^39,48^. Almost the same results were reported by Xiaojia He et al. (2016), the Zeta potential larger than—25 of TA@TiO_2_ NPs^49^.

### Surface morphology

**Figure 6:**
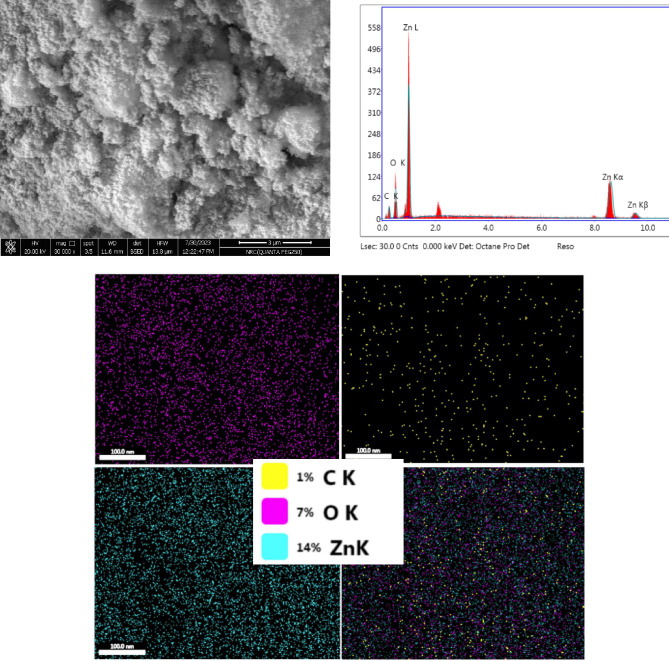
SEM images, EDAX spectrum of ZnO@TA NPs and mapping image.

Figure 6 displays SEM image of ZnO@TA as well as mapping and EDAS data. ZnO@TA showed spherical shape in clusters. The existence of ZnO NPs with homogenous distribution in ZnO@TA is further supported by mapping images. In EDAX spectrum, the proportional elemental composition of ZnO nanoparticles in ZnO@TA was confirmed using the energy dispersive X-rays Analysis (EDAX) tool, as illustrated in Figure 6, by measuring the intensity of the characteristic emitted X- rays. In synthesized ZnO@TA nanoparticles, EDAX revealed only the presence of three elements: carbon, zinc and oxygen. The atomic percent compositions of elements are 59.25%, 38.52%, and 2.23% for C, O, and Zn respectively^50^.

**Figure 7:**
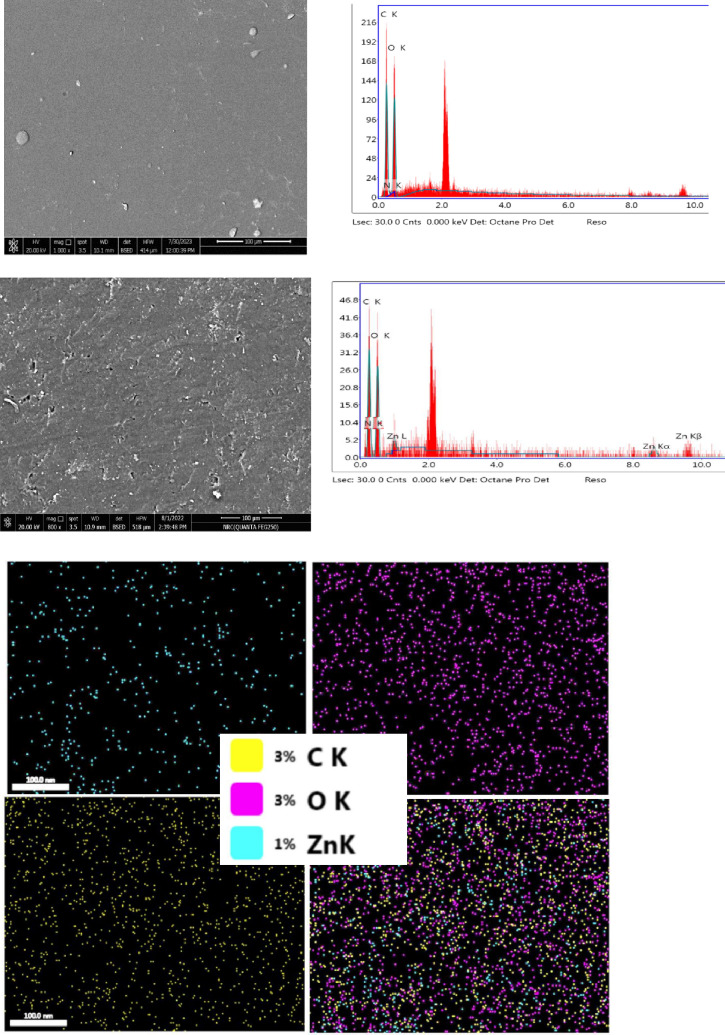
SEM, EDAX, mapping of CH/PVA and ZnO@TA@CH/PVA films.

Figure 7 shows SEM, EDAX, mapping images of CH/PVA and ZnO@TA@CH/PVA films. PVA/CH film shows uniform smooth surface, without any fracture and air bubbles. The surface image of ZnO@TA@CH/PVA film exhibits surface roughness due to inclusion of ZnO@TA NPs. The proportional elemental composition of CH/PVA film shows three elements namely, C, O, and N with atomic percent 49.89%, 44.41%, and 5.7%, respectively. However, the proportional elemental composition of ZnO@TA@CH/PVA film was 55.27%, 36.97%, 5.7%, 2.06% for C, O, N, and Zn, respectively. The mapping images of the film reveal that ZnO@TA nanoparticles are uniformly distributed in ZnO@TA@CH/PVA film matrix as demonstrated in Fig. 7.

### Barrier properties

Table 2 demonstrates the impact of changing ZnO@TA concentrations on barrier properties. WVP measures the rate of moisture that crosses the film, being an important property to be accounted for in packaging applications^51^.

**Table 2:** Influence of ZnO@TA concentrations (mg) WVP and OP of ZnO@TA@CH/PVA film.

Treatments	WVP (g.mm/m^2^.kPa.day)	OP (c.c/m^2^ day)
CH/PVA	43.52 ± 1.01^a^	0.641 ± 1.50 × 10^−2a^
ZnO@TA(10)@CH/PVA	37.93 ± 1.05^b^	0.358 ± 1.00 × 10^−1b^
ZnO@TA(30)@CH/PVA	35.69 ± 1.87^b^	0.246 ± 1.29 × 10^−1c^
ZnO@TA(50)@CH/PVA	31.98 ± 1.68^c^	0.144 ± 5.03 × 10^−2d^

Superscript letters a–d: different superscripts within the same column indicate significant differences among samples (*p* < 0.05).

As expected, WVP of ZnO@TA@CH/PVA films tend to diminish with the incorporation of ZnO@TA NPs compared with CH/PVA control film which is generally explained by the physical crosslinking of ZnO@TA NPs nanocomposite, which will diminish the diffusion of water vapor and gases^33^.

The WVP of ZnO@TA@CH/PVA films shows a significant decrease with increasing ZnO@TA concentrations (*p˂*0.05) in comparison with CH/PVA control. There is no significance difference between PVA/CH films containing ZnO@TA concentrations of 10 and 30 mg however, WVP of ZnO@TA@CH/PVA film containing 50 mg significantly differ than the other films. May be at that concentration (50 mg) is sufficient to cause more crosslinking to CH/PVA films thus restrict mobility of the polymer chains and consequently decreasing the transport of gases. The lowest WVP is 31.98 ± 1.68 g.mm/m^2^.kPa.day which related to ZnO@TA(50)@CH/PVA compared with PVA/CH control film 43.52 ± 1.01 g.mm/m^2^.kPa.day as typed in Table 1.

The O_2_ gas barrier property is another significant factor influencing shelf life of fresh packed food. The oxygen permeability of ZnO@TA@CH/PVA film exhibits a significant decrease in OP (*p* < 0.05). The decreasing sequence of OP is 0.641 ± 1.50 × 10^–2^, 0.358 ± 1.00 × 10^–1^, 0.246 ± 1.29 × 10^–1^, and 0.144 ± 5.03 × 10^–2^ c.c/m^2^ day corresponding to CH/PVA, ZnO@TA(10)@CH/PVA, ZnO@TA(30)@CH/PVA, and ZnO@TA(50)@CH/PVA, respectively as shown in Table 2. This drastic decreasing behavior could be due to crosslinking reasons as mentioned before. Furthermore, ZnO@TA nanoparticles have the potential to develop tortuous pathways that significantly reduce the size of matrix pore channels^52^.

Other researchers have previously documented the same trend, a study reported by Song et al. (2023) indicated that ZnO/plant polyphenols/cellulose/polyvinyl alcohol films showed better water vapor barrier properties than PVA, with a WVP of 12.7 g m^−2^ h^−1^ and 10.8 to 7.5 g m^−2^ h^−1^^53^. In another study worth mentioning in which chitosan and tannic acid were crosslinked in neutral and mildly basic circumstances to create chitosan-tannic acid composite films. The pristine chitosan film had strong transmission rates, as evidenced by its WVTR and OTR of 956 g/m^2^⋅day and 0.39 cc/m^2^⋅day, respectively. The chitosan-tannic acid composite films, on the other hand, exhibited noticeably lower transmission rates than the native chitosan film because of the formation of a denser structure as a result of physical crosslinks between the two substances^19^.

### Solubility of ZnO@TA@CH/PVA film

**Figure 8:**
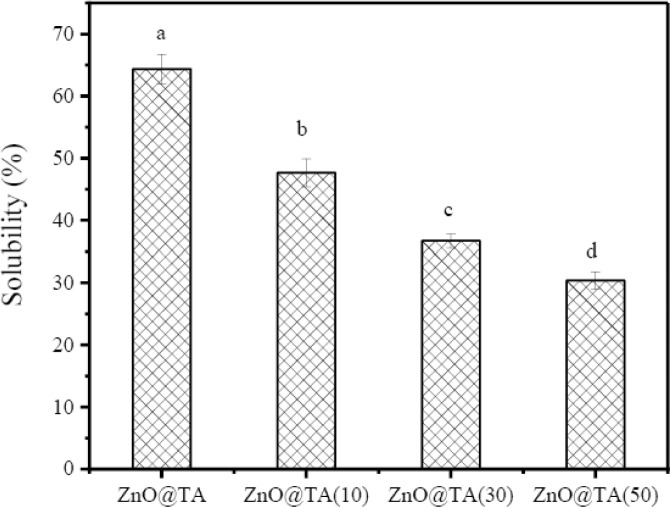
Effect of ZnO@TA changing concentrations on ZnO@TA@ CH/PVA film solubility. a–d: different superscript letters represent significant difference at 5% level of probability (*P* < 0.05).

The influence of ZnO@TA changing concentrations on ZnO@TA@CH/PVA film solubility is shown in Fig. 8. The changing concentration of ZnO@TA has significant impact on solubility of the films (*P* < 0.05). The solubility of ZnO@TA@CH/PVA films drastically tend to decrease with increasing loadings of ZnO@TA. The solubility percentages are 64.35 ± 2.4 %, 47.65 ± 2.2 %, 36.68 ± 1.1%, and 30.36 ± 1.4% that are belonged to CH/PVA, ZnO@TA(10)@CH/PVA, ZnO@TA(30)@CH/PVA, and ZnO@TA(50)@CH/PVA films. These results may be ascribed to crosslinking reasons and development of more hydrogen bonding between ZnO@TA and functional groups of base film components as mentioned before.

### Water contact angles measurements (WCA)

**Figure 9:**
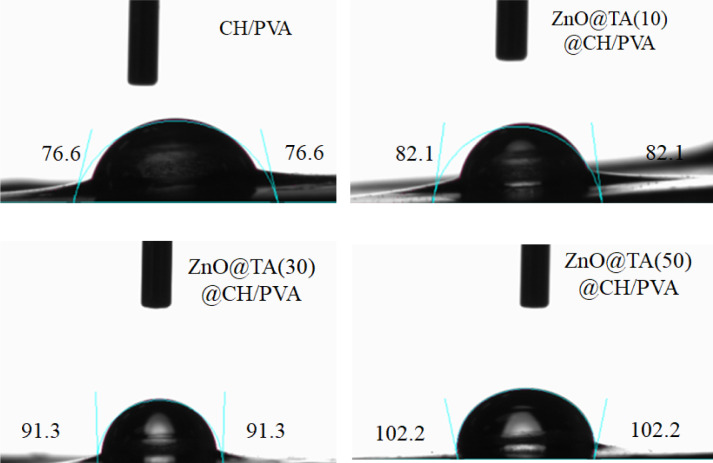
Water contact angles images of ZnO@TA@PVA/CH films.

Figure 9, shows the WCA of CH/PVA and ZnO@TA@CH/PVA films containing different concentrations of ZnO@TA. According to a general theory, a contact angle that is smaller (below 90°) indicates a material's hydrophilic character, whereas a greater value (above 90°) shows a material's hydrophobic nature. The contact angle of CH/PVA film is 76.6°. For ZnO@TA concentrations of 10, 30, and 50 mg, respectively, the contact angles of ZnO@TA@CH/PVA films are increased by 7.18%, 19.19%, and 33.42%, respectively. These findings may due to ZnO@TA addition-induced roughness to the surface of ZnO@TA@CH/PVA films occurred.

### Mechanical properties

Table 3 depicts tensile strength and elongation % at break of ZnO/TA@ PVA/CH as a function of increasing ZnO@TA concentrations. The tensile strength of neat PVA/CH film was 30.47 ± 0.96 MPa. As the loading of ZnO@TA increase, tensile strength of ZnO@TA @PVA/CH composite films significantly increases (*p* < 0.05). Tensile strength of ZnO@TA @PVA/CH composite films are 37.47 ± 1.09, 43.33 ± 1.21, and 48.72 ± 0.23 MPa up to 10, 30, and 50 mg of ZnO@TA loadings, respectively. That enhancement in tensile strength may be attributable to stronger intermolecular forces that can occur between the polymer chains (PVA/CH) and ZnO@TA. When hydroxyl groups of PVA/CH and ZnO@TA nanoparticles interact, covalent and hydrogen bonds developed due to presence of extensive tannic acid hydroxyl moiety, thereby increasing the molecular force. The development of cross links between polymer chains was facilitated by the ZnO@TA. Therefore, films containing ZnO@TA nanoparticles had a more compact film matrix structure, which resulted in the development of stronger films^16,19^. This tensile enhancement is agreed with results reported by Aswathy Jayakumar et al. (2023), active and intelligent composite films based on polyvinyl alcohol, chitosan, zinc oxide nanoparticles, and sweet purple potato extract. The tensile strength of the chitosan/polyvinyl film was 13.0 MPa and increased to 30.8 MPa for chitosan/polyvinyl film composite film containing zinc oxide nanoparticles, and sweet purple potato extract^54^.

**Table 3:** Influence of ZnO@TA concentrations (mg) tensile strength and elongation % at break of ZnO@TA@PVA/CH film.

Treatments	Tensile strength (MPa)	Young’s modulus	Elongation % at break
PVA/CH	30.47 ± 0.96^a^	1034.32 ± 52.8^a^	35.10 ± 2.3^a^
ZnO@TA(10)@PVA/CH	37.47 ± 1.09^b^	1436.66 ± 47.5^b^	32.54 ± 1.07^ab^
ZnO@TA(30)@PVA/CH	43.33 ± 1.21^c^	1905.52 ± 29.3^c^	27.02 ± 2.19^c^
ZnO@TA(50)@PVA/CH	48.72 ± 0.23^d^	2163.46 ± 61.4^d^	19.62 ± 2.3^d^

a–d: different superscripts within the same column indicate significant differences among samples (*p* < 0.05).

Youngs modulus of ZnO@TA @PVA/CH composite films significantly increases (*p* < 0.05). Young`s modulus of ZnO@TA @PVA/CH composite films are 1436.66 ± 47.5, 1905.52 ± 29.3, and 2163.46 ± 61.4 MPa up to 10, 30, and 50 mg of ZnO@TA loadings, respectively compared with control 1034.32 ± 52.8 MPa.

Additionally, table 3 shows the effects of ZnO@TA changing loadings on the elongation at break of PVA/CH composite films. As can be seen, the percentage of elongation at break significantly decreased with increasing of ZnO@TA concentration (*p* < 0.05). Despite the increased increments, there is no significant difference between control film and PVA/CH containing 10 mg ZnO@TA. PVA/CH film showed an elongation at break of 35.10 ± 2.3%, whereas the nanocomposites with 10, 30 and 50 mg ZnO@TA showed an elongation at break of 32.54 ± 1.07, 27.02 ± 2.19, and 19.62 ± 2.3%**,** respectively. This is because the rigidity of matrix increased by adding ZnO@TA. Moreover, strong interaction between ZnO@TA and PVA/CH chains could restrict chain movements and consequently blocks its ability to flow and reduce its ductility^55^. Further, the significant improvement in tensile strength accompanied with decreasing in elongation at break which can be translated as ZnO@TA NPs addition can behave as an efficient reinforcing agent. Notably, the distribution of nanofillers and their interaction with the polymer matrix are strongly correlated with the reinforcing effect in nanocomposite films^56^. Considering the EDAX image in Fig. 7, the significant tensile improvement can be attributed to the homogeneity dispersion of ZnO@TA NPs within CH/PVA film forming strong interfacial adhesion which minimizes phase separation and allows efficient stress transfer at the interface^57^. Indeed, the addition of ZnO@TA NPs in the CH/PVA film matrix enhances mechanical performance. In 2023, Su Jin Lee and colleagues created a multifunctional chitosan (CH) film that contains 0.5–1.0 weight %of tannic acid (TA). Tris buffer (pH 8.5) and phosphate-buffered saline (CH-TA/P) were used to neutralize the chitosan films. The virgin CH's tensile strength and elongation % at break were 58.1 ± 5.1 MPa and 8.1 ± 1.8%, respectively. On the other hand, CH-TA0.5/P, CH-TA0.5/T, CH-TA1.0/P, and CH-TA1.0/T films had significantly greater tensile strengths at 109.8 ± 4.9, 134.0 ± 5.1, 63.0 ± 8.1, and 112.8 ± 8.2 MPa, respectively^19^.

### Antioxidant activity

**Figure 10:**
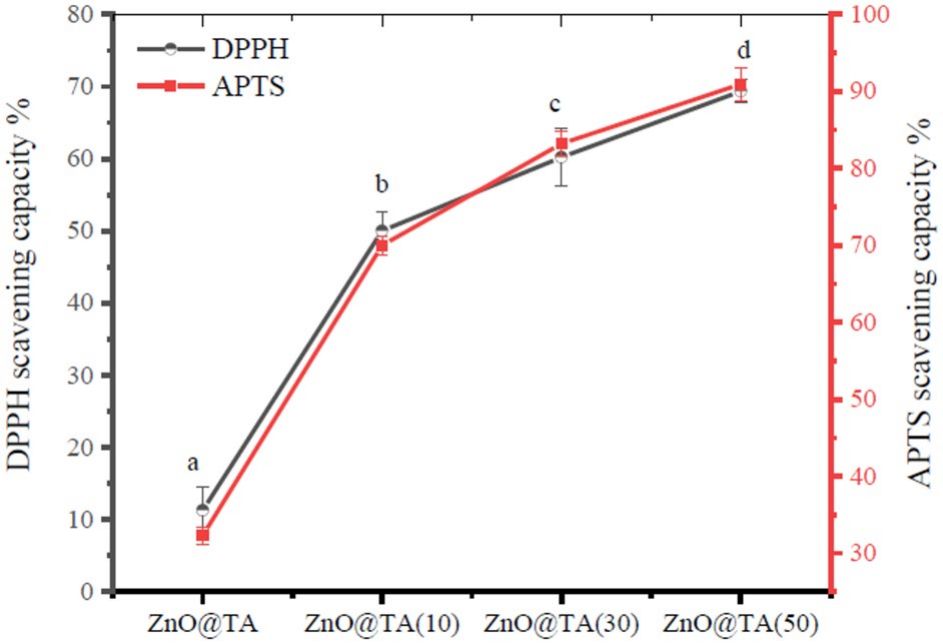
DPPH scavenging ability % of ZnO@TA@PVA/CH films as a function of ZnO@TA concentrations. (**a–h**) Non-identical letters denote statistical difference (*p* < 0.05).

DPPH free radicals scavenging activity, in which DPPH functions as a reducing agent or electron donor, is one of the frequently used techniques for determining antioxidant activity. In the presence of antioxidants, DPPH radicals change from a dark violet color to a transparent color and the absorbance at 517 nm is measured to determine the proportion of DPPH antioxidant activity. Figure 10 depicts the percentage of free radical-scavenging activity of ZnO@TA@PVA/CH films as a function of ZnO@TA concentrations. ZnO@TA@PVA/CH films show a significant increasing in DPPH antioxidant activity in the developed ZnO@TA@PVA/CH films with increasing ZnO@TA when compared control film (*p* < 0.05). The DPPH radical-scavenging activity % of ZnO@TA@PVA/CH films increased gradually to 50.0 ± 2.7%, 60.21 ± 4.0%, and 69.35 ± 1.6%, respectively with the increasing concentration of ZnO@TA from 10 to 50 mg, respectively compared with PVA/CH film (11.29 ± 3.2%). The PVA/CH control film reveals a moderate scavenging activity. However, the increasing in antioxidant activity could be due the galloyl groups in tannic acid may contribute to its potent hydrogen and electron-donating properties^58^. Similarly, Fig. 10 the ABTS radical scavenging ability. The antioxidant activities of CH/PVA, ZnO@TA(10)@CH/PVA, ZnO@TA(30)@CH/PVA, ZnO@TA(50)@CH/PVA films were 32.29 ± 1.1%, 70.0 ± 1.23%, 83.21 ± 1.61%, and 90.95 ± 2.12%, respectively. ZnO@TA@CH/PVA film has superior antioxidant activity towards ABTS more than DPPH radicals. Previous study reports have documented the same phenomenon, a novel antioxidant-containing zinc oxide (ZnO) nanoparticle was created by immobilizing the antioxidant 3-(3,4-dihydroxyphenyl)-2-propenoic acid, also known as caffeic acid, CA), on the surfaces of ZnO nanoparticles treated with micro-dielectric barrier discharge (DBD) plasma. ZnO@CA nanoparticles efficiently scavenged ABTS radicals at concentrations ranging from 20 to 100 µM, with activity ranging from 44.99 to 73.68%, respectively^17^. Also, research has been done on the polyvinyl alcohol-based film's antioxidant capacity when lignin nanoparticles loaded with potassium sorbate (LNP@PS). Pure PVA is not capable of scavenging DPPH. However, the color of the mixture of DPPH and film extracts steadily lightened and eventually turned orange when the ratio of LNP@PS and/or TA added to the composite film increased. This suggested that there was strong antioxidant activity in the composite films. The intensity of the DPPH absorption peak was significantly reduced and the free radical scavenging activity (RSA) increased to 50.9% after only 1% of LNP@PS (LNP@PS-1-TA-0) was added to PVA. With LNP@PS-3-TA-5, the ideal RSA value of 92.6% was attained^59^.

### UV-shielding properties

**Figure 11:**
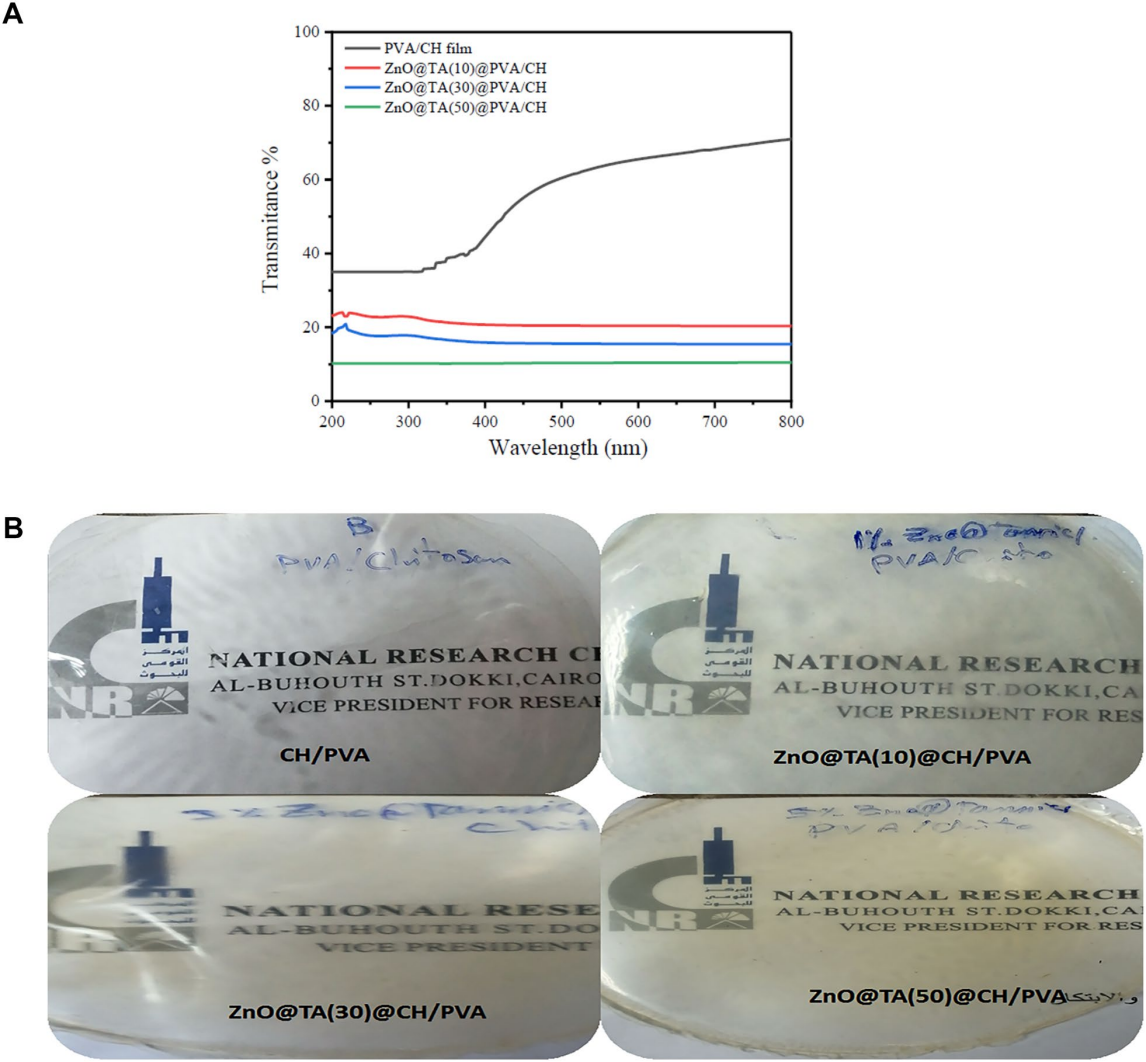
(**A**) UV-shielding properties of CH/PVA and ZnO/TA@CH/PVA films. (**B**) Transparency images of CH/PVA and ZnO/TA@CH/PVA films with different loadings of ZnO@TA.

Food packaging transparency is an essential concern in packaging materials, and it has a clear impact on consumer choices^5,60^. Food shelf life can be reduced by microbial development and/or biochemical processes like oxidation. Exposure to UV light can accelerate lipid oxidation in packaged food, which can cause food to deteriorate. Thus, foods, especially those with high fat content, require antimicrobial packaging materials with potent UV light blocking capabilities property^61^. Although PVA is the most often used packaging material, it has poor UV-shielding qualities. However, PVA UV-shielding performance has been improved by mixing it with biomaterials and nanoparticles^62^ that improve UV absorbance. Furthermore, adding a third phase to the PVA/biomaterials mixture, such as nanomaterials filler, could improve the UV-blocking properties^9,63^. The optical characteristics of the PVA/CH and ZnO@TA @PVA/CH films have been assessed using digital images Fig. 11A. As the ZnO@TA content increased, all of the ZnO@TA @PVA/CH films turned pale brown. Figure 11B depicts the UV transmittance of neat PVA/CH and ZnO@TA @PVA/CH films using various ZnO@TA NP ratios which illustrate the effect of ZnO@TA NP ratios on the UV-shielding properties of the PVA/ CH films. Corresponding to the equations the transmittance of UV-A (320–400) and UV-B (280–320) were implemented to explore UV Shielding properties. The virgin PVA/CH film exhibits an excellent UV shielding capacity where UV transmittance of UVA (91.414%) and UVB (99.198%) and that can be attributed to hydrogen bonding as a HOMO–LUMO interaction between PVA and CH.

On the other side, the incorporation of ZnO@TA NP on PVA/CH film shows an increase in the UV shielding capacity with the ratio increasing where UVA (98.58518%) and UVB (99.21196%) of 10% ZnO@TA @PVA/CH, UVA (98.56131%) and UVB (99.56156%) of 30% ZnO@TA@PVA/CH, and UVA (99.959%) and UVB (99.994%) of 50% ZnO@TA@PVA/CH. This is due to π → π* transition and locally excited π_i_ → π_j_* transition in benzene rings of TA, σ → σ* between O–Zn–O and hydrogen bonding as a HOMO–LUMO interaction between PVA-TA-CH.

Multiple investigators have already documented similar behaviors, for example, the biocomposite ZnO/plant polyphenols/cellulose/polyvinyl alcohol film was created. According to the light shielding study, the cellulose/PVA film contained 1wt% of ZnO/polyphenol mixture can virtually completely filter UV and visible light^53^. In a nother investigation, lignin nanoparticles loaded with potassium sorbate (LNP@PS) as additives to polyvinyl alcohol-based active packaging films have been explored. Pure PVA films show terrible UV-shielding performance but outstanding optical transparency, with transmittance ratios of 80%, particularly in the UVA and UVB regions, and 90% in the visible light range. The UV shielding performance increased from 96.78 to 989.99% when comparing film containing 3% LNP@PS/3% TA and 3% LNP@PS/5%TA, and from 94.79 to 989.99% when comparing with film containing 1% LNP@PS/5%TA and 3% LNP@PS/5%TA, respectively. Both showed a notable decrease in UV transmittance^59^.

### Thermal decomposition analysis studies

**Figure 12:**
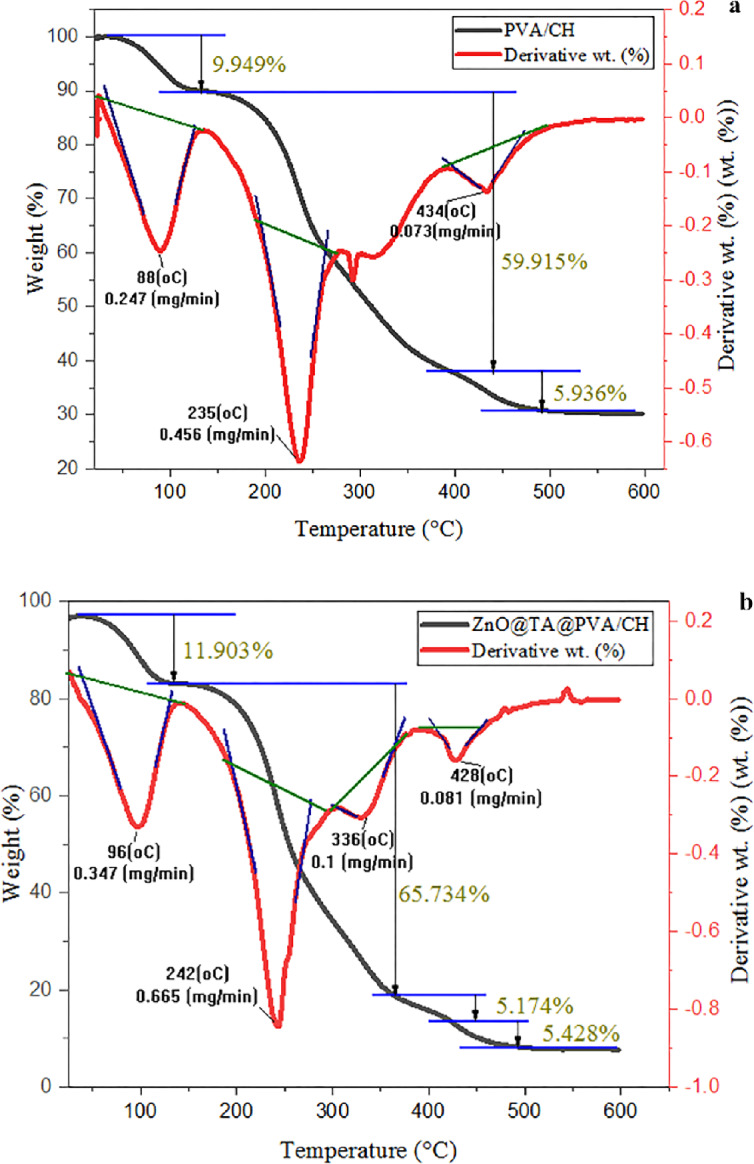
TGA profile of (**a**) PVA/CH and (**b**) ZnO@TA @PVA/CH films.

The thermodynamic properties of CH/PVA and ZnO@TA@CH/PVA composite films were investigated using thermogravimetric analysis, as illustrated in Fig. 12. The initial weight loss for all the produced films took place between 25 and 130 °C, which might be attributable to the loss of absorbed water molecules, notably the weight loss increased after adding ZnO@TA attributed to the degradation of tannins small molecules such as CO, CO_2_, and phenol from high molecular weight macromolecules into smaller chain fragments^64^. The initial decomposition temperature of ZnO@TA @CH/PVA composite film was slightly elevated to 242 °C when contrasted with CH/PVA film (235 °C) with weight loss percentage. The rise in early degradation temperature could be related to the inclusion of ZnO@TA nanoparticles as shown in Table 5.

CH/PVA film exhibits three-stage decomposition however, ZnO@TA @CH/PVA film has four degradation steps. The major second thermal decomposition step for CH/PVA is in the temperatures range 136.45–278.14 °C was attributed to the thermal decomposition of chitosan by deacetylation^4^. ZnO@TA @CH/PVA film sifts to higher temperature range (142.54–304.0 °C) and that may be attributed to the thermal stability of ZnO@TA@CH/PVA film films being better than CH/PVA film after addition of ZnO@TA NPs (Table 4). This temperature range is linked to the dehydration of the saccharide rings, depolymerisation, and decomposition of the polymer units^65^.

**Table 4:** Thermal degradation kinetic parameters of CH/PVA and ZnO@TA @CH/PVA films.

Step	Temp. range °C	Max. weight loss °C	*R*^2^	*RSS*	*n*	*E*_a_ (K J mol^−1^)
PVA/CH						
1st	25.0–130.82	88	0.9995	2.08 × 10^−13^	0.5	− 0.0118
2nd	136.45–278.14	235	0.9917	1.32 × 10^−12^	0.5	− 0.0102
3th	391.72–489.43	434	0.9954	1.56 × 10^−14^	0.5	− 0.0060* Ʃ *E*_a_ − 0.02803
ZnO@TA @PVA/CH						
1st	25.19–138.21	96	0.9993	3.52 × 10^–3^	0.5	− 0.01147
2nd	142.54–304.0	242	0.9908	1.71 × 10^–12^	0.5	− 0.01039
3th	304.65–373.79	336	0.9951	1.72 × 10^–14^	0.5	− 0.00796
4th	404.03–608.02	428	0.9965	2.44 × 10^–15^	0.5	− 0.00637* Ʃ *E*_a_ − 0.03620

*Significant values.

And the maximum weight loss rate temperature was slightly increased with the addition of ZnO@TA content from 42.77 wt % to 67.0 wt.% owing to the strong interfacial interactions between the functional groups of ZnO@TA and the macromolecular chains of CH/PVA.

The thermal decomposition of PVA as per previous report^66,67^ occurred between 391.72 and 489.43 °C and 404.03–459.97 °C for CH/PVA and ZnO@TA @CH/PVA respectively. The third decomposition range of ZnO@TA @CH/PVA is between 303.65 and 373.79 °C which could be attributed to two reasons the first is the depolymerisation/hydrolysis of tannic acid^64^ and the second is the thermal degradation of cross-linked bond (hydrogen bond) forming via tannins polyols occurred with PVA and chitosan beside to the degradation of zinc oxide nanoparticles of ZnO@TA @CH/PVA film (Table 4). Previous research has shown that zinc oxide nanoparticles can operate as thermal insulators by limiting the mobility of polymer chains. This demonstrated the thermal stability of the generated composite films and was consistent with prior results^68^. The knowledge of *E*_*a*_ allows detecting reaction mechanism over a wide temperature range. The total activation energy (Ʃ *E*_a_) of ZnO@TA @CH/PVA is higher than CH/PVA. The Ʃ *E*_a_ are −0.02803 K J mol−^1^ and −0.03620 K J mol^−1^ for CH/PVA and ZnO@TA@CH/PVA, respectively (Table 4). This result indicates that ZnO@TA @CH/PVA film is more thermally stable than CH/PVA film.

### Antimicrobial studies

**Figure 13:**
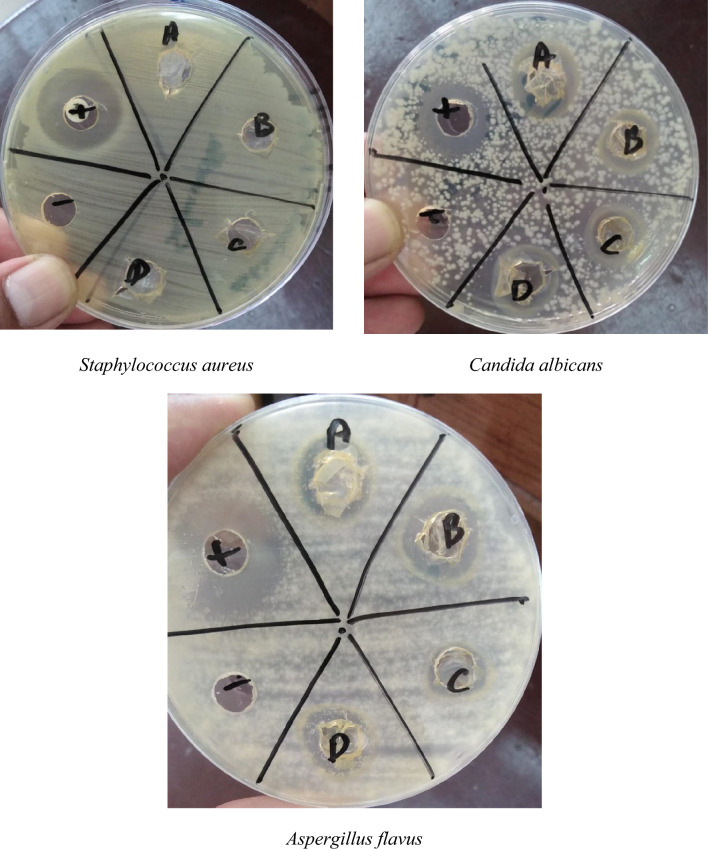
Inhibition zones generated by C: CH/PVA control film, D: ZnO@TA (10 mg)@PVA/CH, B: ZnO@TA (30 mg)@PVA/CH, and A: ZnO@TA(50 mg)@PVA/CH.

Figure 13 shows the antimicrobial activity against Gram-Positive Bacteria: *Staphylococcus aureus, pathogenic yeast Candida albicans and* crops pathogen*: Aspergillus flavus.* All films show antimicrobial influence against all tested microorganisms. As shown in Table 5, the inhibition zones (mm) significantly increase with increasing loadings of ZnO@TA, the effective concentration of ZnO@TA is 50 mg at which ZnO@TA@CH/PVA film exhibits the largest inhibition zone (11 ± 1.0 mm) against *Staphylococcus aureus* compared with amoxicillin/clavulanic positive control (26 ± 1.0 mm) and 12.3 ± 0.57 mm and 13.6 ± 0.57 mm for *Aspergillus flavus and Candida albicans*, respectively compared with clotrimazole fungal positive control 15.0 ± 1.0 mm and 14.6 ± 0.57 mm, respectively. Several studies have demonstrated the efficacy of zinc oxide nanoparticles for bacterial membrane disruption, enzymatic inhibition, interaction with genes and proteins, and protein inactivation^69^.

**Table 5:** Inhibition zones readings caused by CH/PVA and ZnO@TA@PVA/CH films with different loadings of ZnO@TA.

Film	*Staphylococcus aureus*	*Aspergillus flavus*	*Candida albicans*
CH/PVA	5 ± 1.0^a^	6.3 ± 0.57^a^	8.3 ± 0.57^a^
ZnO@TA (10 mg)@PVA/CH	8 ± 1.0^b^	9.8 ± 0.57^b^	9.0 ± 1.0^a^
ZnO@TA (30 mg)@PVA/CH	9 ± 1.0^bc^	10.16 ± 1.4^bc^	11.0 ± 0.57^b^
ZnO@TA (50 mg)@PVA/CH	11 ± 1.0^d^	12.3 ± 0.57^d^	13.6 ± 0.57^c^
Positive control	26 ± 1.0^e^	15.0 ± 1.0^e^	14.6 ± 0.57^cd^

Positive controls: amoxicillin/clavulanic acid 30 µg (*bacterial*) and clotrimazole 1 mg/ml (*fungal*). a–e: non-identical letters in each column denote statistical difference (*p* < 0.05).

Previous studies have shown comparable behavior, Kyong-Hoon Choi et al. (2017) have reported preparation of a new zinc oxide (ZnO) nanoparticle with antioxidant capabilities. The ZnO nanoparticles were treated with micro-dielectric barrier discharge (DBD) plasma to immobilize the antioxidant 3-(3,4-dihydroxyphenyl)-2-propenoic acid (caffeic acid, CA). ZnO@CA nanoparticles shown strong antibacterial action against *Escherichia coli* and *Staphylococcus aureus*, including resistant strains like methicillin-resistant *S. aureus*^17^. Also, cellulose/PVA film containing (1.0 wt% zinc oxide/plant polyphenols was created by Da Song and Li-Wei Ma et al. (2023) through straightforward hydrothermal and casting techniques. The study evaluated the antibacterial activities of against *Escherichia coli* and *staphylococcus aureus*, achieving 4.4 and 6.3 mm inhibition zones, respectively^53^. A study has been reported by Denice S. Vicentini et al. (2010) has prepared ZnO nanoparticles have been produced from polyester using the Pechini method, which involves reacting citric acid and ethylene glycol. The resulting ZnO nanoparticles were then mixed with varying concentrations of polyoxyethylene sorbitan monooleate, or Tween 80 (T80), to create blend films of chitosan (CH) and poly (vinyl alcohol) (PVA). When the films' antibacterial activity was evaluated, the ZnO nanoparticle-containing films demonstrated antibacterial activity against the bacterium species *Staphylococcus aureus*. Without ZnO nanoparticles added, the *S. aureus* species microorganisms in the films continued to be viable; but, when exposed to ZnO nanoparticles, they ceased to be viable. Consequently, these findings imply that the ZnO nanoparticles' presence is what causes the antibacterial activity^11,70^. Another study examined the efficiency of chitosan-coated film with varied quantities of *Moringa oleifera* seed powder (MOSP) as reinforcement agent and tannic acid (TA) as a crosslinker reported by Raja Venkatesan et al. (2024). The biocomposite films, with 10.0 wt.% MOSP content, showed increased antimicrobial and antifungal activity against bacteria like *Staphylococcus aureus*, *E. coli*, *A. niger*, and *Candida albicans*, making them ideal food packaging materials^71^.

## Conclusion

Active packaging film ZnO@TA@PVA/CH with different ratios of ZnO@TA nanocomposite was successfully created. The structure of the prepared films was characterized using FTIR, and XRD which revealed the existence of ZnO@TA nanocomposite in the PVA/CH matrix film. Moreover, the morphological structure of ZnO@TA @PVA/CH composite film was figured using TEM, and SEM which showed crystallites characteristic of nanostructured ZnO@TA, in addition. The mapping images of the film reveal that ZnO@TA nanoparticles are uniformly distributed in ZnO@TA@PVA/CH film matrix. The results also showed that increasing ZnO@TA content enhanced the thermal stability, water vapor barrier properties, antibacterial and mechanical properties of PVA/CH films. Besides, increasing ZnO@TA nanocomposite content showed a significant increase in DPPH antioxidant activity and UV-Shielding properties. ZnO@TA @PVA/CH film has a broad antimicrobial spectrum action against Gram-Positive Bacteria: *Staphylococcus aureus, pathogenic yeast Candida albicans and* crops pathogen*: Aspergillus flavus.* The maximum inhibition zone was created by ZnO@TA(50)@PVA/CH film. In light of the previous results, incorporating ZnO@TA nanocomposite in the PVA/CH matrix could be a promising way for efficient biodegradable packaging material.

The demands of the modern world cannot be adequately and successfully met by traditional packaging. To reduce pathogens resistance in food, active packaging will grow in the future due to consumer preferences minimally processed and naturally preserved products as well as the food industry's need for significant investments in the quality and safety of its products. Additional research is required in a number of active-packaging system domains for this purpose. To improve film characteristics, future studies should look at large-scale production and the incorporation of additives such natural extracts and nanoparticles. Films made of a chitosan/PVA combination provide a viable, environmentally friendly substitute for plastic packaging manufactured from petroleum, and they may find use in actual food preservation.

## Data Availability

All data generated or analyzed during this study are included in this article.

## References

[1] Ezati, P. & Rhim, J.-W. Fabrication of quercetin-loaded biopolymer films as functional packaging materials. *ACS Appl. Polymer Mater.* **3**(4), 2131–2137 (2021). 10.1021/acsapm.1c00177

[2] Alizadeh Sani, M. *et al.* Intelligent packaging systems for the quality and safety monitoring of meat products: From lab scale to industrialization. *Food Control* **160**, 110359 (2024). 10.1016/j.foodcont.2024.110359

[3] Mondal, K. *et al.* Development of antioxidant-rich edible active films and coatings incorporated with de-oiled ethanolic green algae extract: A candidate for prolonging the shelf life of fresh produce. *RSC Adv.* **12**(21), 13295–13313 (2022). 35520137 10.1039/D2RA00949H PMC9062619

[4] Liu, Y., Wang, S. & Lan, W. Fabrication of antibacterial chitosan-PVA blended film using electrospray technique for food packaging applications. *Int. J. Biol. Macromol.* **107**, 848–854 (2018). 28923566 10.1016/j.ijbiomac.2017.09.044

[5] Hu, W. *et al.* Fabrication of highly transparent and multifunctional polyvinyl alcohol/starch based nanocomposite films using zinc oxide nanoparticles as compatibilizers. *Int. J. Biol. Macromol.* **204**, 284–292 (2022). 35149089 10.1016/j.ijbiomac.2022.02.020

[6] Oussalah, M. *et al.* Antimicrobial and antioxidant effects of milk protein-based film containing essential oils for the preservation of whole beef muscle. *J. Agric. Food Chem.* **52**, 5598–5605 (2004). 15373399 10.1021/jf049389q

[7] Sultan, M. *et al.* Active packaging of chitosan film modified with basil oil encapsulated in silica nanoparticles as an alternate for plastic packaging materials. *Food Biosci.* **51**, 102298 (2023). 10.1016/j.fbio.2022.102298

[8] Zhang, W. *et al.* Advances in sustainable food packaging applications of chitosan/polyvinyl alcohol blend films. *Food Chem.* **443**, 138506 (2024). 38306905 10.1016/j.foodchem.2024.138506

[9] Oun, A. A. *et al.* Recent advances in polyvinyl alcohol-based composite films and their applications in food packaging. *Food Packag. Shelf Life* **34**, 100991 (2022). 10.1016/j.fpsl.2022.100991

[10] Oun, A. A., Shin, G. H. & Kim, J. T. Antimicrobial, antioxidant, and pH-sensitive polyvinyl alcohol/chitosan-based composite films with aronia extract, cellulose nanocrystals, and grapefruit seed extract. *Int. J. Biol. Macromol.* **213**, 381–393 (2022). 35654221 10.1016/j.ijbiomac.2022.05.180

[11] Vicentini, D. S., Smania, A. & Laranjeira, M. C. M. Chitosan/poly (vinyl alcohol) films containing ZnO nanoparticles and plasticizers. *Mater. Sci. Eng. C* **30**(4), 503–508 (2010). 10.1016/j.msec.2009.01.026

[12] Alizadeh Sani, M., Khezerlou, A. & McClements, D.J. Zeolitic imidazolate frameworks (ZIFs): Advanced nanostructured materials to enhance the functional performance of food packaging materials. *Adv. Colloid Interface Sci*. **327**, 103153 (2024). 10.1016/j.cis.2024.103153 38604082

[13] Anean, H. A. *et al.* Nano edible coatings and films combined with zinc oxide and pomegranate peel active phenol compounds has been to extend the shelf life of minimally processed pomegranates. *Materials.*10.3390/ma16041569 (2023). 36837201 10.3390/ma16041569 PMC9965157

[14] Dulta, K. *et al.* Development of alginate-chitosan based coating enriched with ZnO nanoparticles for increasing the shelf life of orange fruits (*Citrus sinensis* L.). *J. Polymers Environ.* **30**(8), 3293–3306 (2022). 10.1007/s10924-022-02411-7

[15] Souza, V. G. *et al.* Biodegradable chitosan films with ZnO nanoparticles synthesized using food industry by-products—Production and characterization. *Coatings*10.3390/coatings11060646 (2021). 10.3390/coatings11060646

[16] Lee, J. *et al.* Functionalized ZnO nanoparticles with gallic acid for antioxidant and antibacterial activity against methicillin-resistant *S. aureus*. *Nanomaterials* **7**, 365 (2017). 29099064 10.3390/nano7110365 PMC5707582

[17] Choi, K.-H. *et al.* Antioxidant potential and antibacterial efficiency of caffeic acid-functionalized ZnO nanoparticles. *Nanomaterials*10.3390/nano7060148 (2017). 28621707 10.3390/nano7060148 PMC5485795

[18] Halim, A. L. A., Kamari, A. & Phillip, E. Chitosan, gelatin and methylcellulose films incorporated with tannic acid for food packaging. *Int. J. Biol. Macromol.* **120**, 1119–1126 (2018). 30176328 10.1016/j.ijbiomac.2018.08.169

[19] Lee, S. J. *et al.* Multifunctional chitosan/tannic acid composite films with improved anti-UV, antioxidant, and antimicrobial properties for active food packaging. *Food Hydrocolloids* **136**, 108249 (2023). 10.1016/j.foodhyd.2022.108249

[20] Arefi, M. & Rezaei-Zarchi, S. Synthesis of zinc oxide nanoparticles and their effect on the compressive strength and setting time of self-compacted concrete paste as cementitious composites. *Int. J. Mol. Sci.* **13**, 4340–4350 (2012). 22605981 10.3390/ijms13044340 PMC3344217

[21] Noshirvani, N. *et al.* Novel active packaging based on carboxymethyl cellulose-chitosan-ZnO NPs nanocomposite for increasing the shelf life of bread. *Food Packag. Shelf Life* **11**, 106–114 (2017). 10.1016/j.fpsl.2017.01.010

[22] Yadav, S. *et al.* Preparation, physicochemical and biological evaluation of quercetin based chitosan-gelatin film for food packaging. *Carbohyd. Polym.* **227**, 115348 (2020). 10.1016/j.carbpol.2019.115348 31590881

[23] Souza, V. *et al.* Biodegradable chitosan films with ZnO nanoparticles synthesized using food industry by-products-production and characterization. *Coatings* **11**, 31 (2021). 10.3390/coatings11060646

[24] Ji, M. *et al.* Physical properties and bioactivities of fish gelatin films incorporated with cinnamaldehyde-loaded nanoemulsions and vitamin C. *LWT* **135**, 110103 (2020). 10.1016/j.lwt.2020.110103

[25] Baliyan, S. *et al.* Determination of antioxidants by DPPH radical scavenging activity and quantitative phytochemical analysis of *Ficus religiosa*. *Molecules* **27**, 11 (2022). 10.3390/molecules27041326 PMC8878429 35209118

[26] Sultan, M., Hafez, O. M. & Saleh, M. A. Active coating film based on gelatin/trans-cinnamic acid for cold preservation of peach fruits (*Prunus persica* L. Bastch). *J. Stored Prod. Res.* **106**, 102286 (2024). 10.1016/j.jspr.2024.102286

[27] Sultan, M. *et al.* Active packaging gelatin films based on chitosan/Arabic gum/coconut oil Pickering nano emulsions. *J. Appl. Polymer Sci.* **139**(1), 51442 (2022). 10.1002/app.51442

[28] Abdel Baseer, R. *et al.* A biodegradable film based on cellulose and thiazolidine bearing UV shielding property. *Sci. Rep.* **12**, 33 (2022). 35550531 10.1038/s41598-022-11457-5 PMC9098501

[29] Zhang, X.-F. *et al.* Highly transparent graphene oxide/cellulose composite film bearing ultraviolet shielding property. *Int. J. Biol. Macromol.* **3**, 145 (2019). 10.1016/j.ijbiomac.2019.12.241 31891698

[30] Tohamy, H.-A.S., Taha, G. & Sultan, M. Dialdehyde cellulose/gelatin hydrogel as a packaging material for manganese oxides adsorbents for wastewater remediation: Characterization and performance evaluation. *Int. J. Biol. Macromol.* **248**, 125931 (2023). 37481186 10.1016/j.ijbiomac.2023.125931

[31] Sultan, M. *et al.* Fabrication and evaluation of antimicrobial cellulose/Arabic gum hydrogels as potential drug delivery vehicle. *Int. J. Biol. Macromol.* **242**, 125083 (2023). 37247718 10.1016/j.ijbiomac.2023.125083

[32] Ghanem, A. F. *et al.* Enhancement the photocatalytic and biological activity of nano-sized ZnO using hyperbranched polyester. *J. Inorgan. Organomet. Polymers Mater.* **29**(3), 928–938 (2019). 10.1007/s10904-018-01067-y

[33] Yadav, S., Mehrotra, G. & Dutta, P. Chitosan based ZnO nanoparticles loaded gallic-acid films for active food packaging. *Food Chem.* **334**, 127605 (2020). 32738726 10.1016/j.foodchem.2020.127605

[34] Jayachandran, A. & Nair, A. Green synthesis and characterization of zinc oxide nanoparticles using *Cayratia pedata* leaf extract. *Biochem. Biophys. Rep.* **26**, 100995 (2021). 33898767 10.1016/j.bbrep.2021.100995 PMC8055550

[35] Jayarambabu, N. Germination and growth characteristics of mungbean seeds (*Vigna radiata* L.) affected by synthesized zinc oxide nanoparticles. *Int. J. Curr. Eng. Technol.* **4**, 5 (2014).

[36] Jayarambabu, N. *et al.* Beneficial role of zinc oxide nanoparticles on green crop production. *Int. J. Multidiscip. Adv. Res. Trends* **2**, 2349–7408 (2015).

[37] Costa, G. *et al.* Preparation, characterization and antioxidant evaluation of Cu(II) and Zn(II) tannates. *Open Chem. J.* **5**, 158–171 (2018). 10.2174/1874842201805010158

[38] Wahyono, T. *et al.* Fourier transform mid-infrared (FTIR) spectroscopy to identify tannin compounds in the panicle of *Sorghum* mutant lines. *IOP Conf. Ser. Mater. Sci. Eng.* **546**, 042045 (2019). 10.1088/1757-899X/546/4/042045

[39] Chen, C. *et al.* Tannic acid: A crosslinker leading to versatile functional polymeric networks: A review. *RSC Adv.* **12**(13), 7689–7711 (2022). 35424749 10.1039/D1RA07657D PMC8982347

[40] Melo-Silveira, R. *et al.* In vitro antioxidant, anticoagulant and antimicrobial activity and in inhibition of cancer cell proliferation by xylan extracted from corn cobs. *Int. J. Mol. Sci.* **13**, 409–426 (2012). 22312261 10.3390/ijms13010409 PMC3269695

[41] Queiroz, M. *et al.* Does the use of chitosan contribute to oxalate kidney stone formation?. *Mar. Drugs* **13**, 141–158 (2014). 25551781 10.3390/md13010141 PMC4306929

[42] Tymczewska, A. *et al.* Functional properties of gelatin/polyvinyl alcohol films containing black cumin cake extract and zinc oxide nanoparticles produced via casting technique. *Int. J. Mol. Sci.*10.3390/ijms23052734 (2022). 35269873 10.3390/ijms23052734 PMC8911258

[43] Abdeen, Z., Mohammad, S. G. & Mahmoud, M. S. Adsorption of Mn (II) ion on polyvinyl alcohol/chitosan dry blending from aqueous solution. *Environ. Nanotechnol. Monit. Manag.* **3**, 1–9 (2015).

[44] Gasti, T. *et al.* Chitosan/pullulan based films incorporated with clove essential oil loaded chitosan-ZnO hybrid nanoparticles for active food packaging. *Carbohydr. Polymers* **277**, 118866 (2022). 10.1016/j.carbpol.2021.118866 34893271

[45] Jalal, R. *et al.* ZnO nanofluids: Green synthesis, characterization, and antibacterial activity. *Mater. Chem. Phys.* **121**(1), 198–201 (2010). 10.1016/j.matchemphys.2010.01.020

[46] Kamaruzaman, A. & Lah, N. A. C. Formation of ZnO nanoparticles in the presence of Tannic acid. *Mater. Today Proc.* **41**, 61–64 (2021). 10.1016/j.matpr.2020.11.1007

[47] Gemta, A., Kim, H. & Hwang, Y.-H. Probing the interaction of caffeic acid (CFA) with ZnO nanoparticles (NPs). *Luminescence* **4**, 31 (2015). 10.1002/bio.3007 27037967

[48] Mojtabataghizadeh, S. & Safaei Javan, R. Preparation and Investigation of chitosan nanoparticles including salicylic acid as a model for an oral drug delivery system. *E-Polymers* **10**, 4 (2010). 10.1515/epoly.2010.10.1.370

[49] He, X. *et al.* Assessing the effect of different natural dissolved organic matters on the cytotoxicity of titanium dioxide nanoparticles with bacteria. *J. Environ. Sci.* **48**, 230–236 (2016). 10.1016/j.jes.2016.02.012 27745668

[50] Nagarajan, S. & Arumugam Kuppusamy, K. Extracellular synthesis of zinc oxide nanoparticle using seaweeds of Gulf of Mannar, India. *J. Nanobiotechnol.* **11**(1), 39 (2013). 10.1186/1477-3155-11-39 PMC3879036 24298944

[51] Rahman, P. *et al.* Chitosan/nano ZnO composite films: Enhanced mechanical, antimicrobial and dielectric properties. *Arab. J. Chem.* **11**, 4 (2016).

[52] Shankar, S., Wang, L.-F. & Rhim, J.-W. Incorporation of zinc oxide nanoparticles improved the mechanical, water vapor barrier, UV-light barrier, and antibacterial properties of PLA-based nanocomposite films. *Mater. Sci. Eng. C Biomim. Mater. Sens. Syst.* **4**, 289–298 (2018). 10.1016/j.msec.2018.08.002 30274061

[53] Song, D. *et al.* An active bio-based food packaging material of ZnO@Plant polyphenols/cellulose/polyvinyl alcohol: Design, characterization and application. *Int. J. Mol. Sci.*10.3390/ijms24021577 (2023). 36675089 10.3390/ijms24021577 PMC9865695

[54] Jayakumar, A. *et al.* Active and intelligent packaging films based on PVA/chitosan/zinc oxide nanoparticles/sweet purple potato extract as pH sensing and antibacterial wraps. *Food Biosci.* **56**, 103432 (2023). 10.1016/j.fbio.2023.103432

[55] Kord, B. *et al.* Preparation and characterization of nanofibrillated cellulose/poly (vinyl alcohol) composite films. *Maderas Cienc. Tecnol.* **18**, 743–752 (2016).

[56] Roy, S. *et al.* Tannic acid cross-linked and TiO_2_ nanoparticles reinforced chitosan-based nanocomposite film. *Polymers* **13**, 228 (2021). 33440770 10.3390/polym13020228 PMC7826602

[57] Raha, S. & Ahmaruzzaman, M. ZnO nanostructured materials and their potential applications: Progress, challenges and perspectives. *Nanoscale Adv.* **4**(8), 1868–1925 (2022). 36133407 10.1039/D1NA00880C PMC9419838

[58] Ahmed, S. & Janaswamy, S. Strong and biodegradable films from avocado peel fiber. *Indus. Crops Prod.* **201**, 116926 (2023). 10.1016/j.indcrop.2023.116926

[59] Zeng, S. *et al.* Multi-functional polyvinyl alcohol/tannin acid composite films incorporated with lignin nanoparticles loaded by potassium sorbate. *Int. J. Biol. Macromol.* **264**, 130474 (2024). 38428769 10.1016/j.ijbiomac.2024.130474

[60] Wen, Y.-H. *et al.* Antibacterial nanocomposite films of poly(vinyl alcohol) modified with zinc oxide-doped multiwalled carbon nanotubes as food packaging. *Polymer Bull.* **79**(6), 3847–3866 (2022). 10.1007/s00289-021-03666-1

[61] Zhang, X. *et al.* Antimicrobial and UV blocking properties of composite chitosan films with curcumin grafted cellulose nanofiber. *Food Hydrocolloids* **112**, 106337 (2021). 10.1016/j.foodhyd.2020.106337

[62] Lai, W.-F., Zhao, S. & Chiou, J. Antibacterial and clusteroluminogenic hypromellose-graft-chitosan-based polyelectrolyte complex films with high functional flexibility for food packaging. *Carbohydr. Polymers* **271**, 118447 (2021). 10.1016/j.carbpol.2021.118447 34364582

[63] Azizi, S. *et al.* Cellulose nanocrystals/ZnO as a bifunctional reinforcing nanocomposite for poly(vinyl alcohol)/chitosan blend films: Fabrication, characterization and properties. *Int. J. Mol. Sci.* **15**, 11040–11053. 10.3390/ijms150611040 (2014). 24945313 10.3390/ijms150611040 PMC4100197

[64] Alam, M. *et al.* Thermal decomposition and kinetic studies of tannic acid using model free-methods. *J. Chilean Chem. Soc.* **63**, 3824–3828 (2018). 10.4067/s0717-97072018000103824

[65] Paulino, A. T. *et al.* Characterization of chitosan and chitin produced from silkworm crysalides. *Carbohydr. Polym.* **64**(1), 98–103 (2006). 10.1016/j.carbpol.2005.10.032

[66] Mathew, S., Nil, J. & Krishnankutty, R. Polyvinyl alcohol/silver nanocomposite films fabricated under the influence of solar radiation as effective antimicrobial food packaging material. *J. Polym. Res.* **26**, 3 (2019). 10.1007/s10965-019-1888-0

[67] Peng, Z. & Kong, L. X. A thermal degradation mechanism of polyvinyl alcohol/silica nanocomposites. *Polym. Degrad. Stabil.* **92**(6), 1061–1071 (2007). 10.1016/j.polymdegradstab.2007.02.012

[68] Vicentini Jr, D. Chitosan/poly (vinyl alcohol) films containing ZnO nanoparticles and plasticizers. *Mater. Sci. Eng. C* **30**, 503–508 (2010). 10.1016/j.msec.2009.01.026

[69] Krishnamoorthy, R. *et al.* Antibacterial mechanisms of zinc oxide nanoparticle against bacterial food pathogens resistant to beta-lactam antibiotics. *Molecules*10.3390/molecules27082489 (2022). 35458685 10.3390/molecules27082489 PMC9032754

[70] Saeed, A. *et al*. Enhancing optical, structural, thermal, electrical properties, and antibacterial activity in chitosan/polyvinyl alcohol blend with ZnO nanorods: Polymer nanocomposites for optoelectronics and food/medical packaging applications. *Polym. Bull*. (2024).

[71] Venkatesan, R. *et al.* Chitosan-based films blended with tannic acid and *Moringa oleifera* for application in food packaging: The preservation of strawberries (*Fragaria ananassa*). *Polymers*10.3390/polym16070937 (2024). 38611195 10.3390/polym16070937 PMC11013215

